# Designing HIV Vaccine Efficacy Trials in the Context of Highly Effective Non-vaccine Prevention Modalities

**DOI:** 10.1007/s12561-020-09292-1

**Published:** 2020-10-22

**Authors:** Holly Janes, Yifan Zhu, Elizabeth R. Brown

**Affiliations:** 1 Fred Hutchinson Cancer Research Center, 1100 Fairview Ave. N., Seattle, WA, USA

**Keywords:** HIV prevention, Clinical trial design, Randomized controlled trial, Vaccine

## Abstract

The evolving HIV prevention landscape poses challenges to the statistical design of future trials of candidate HIV vaccines. Study designs must address the anticipated reduction in HIV incidence due to adding new prevention modalities to the standard prevention package provided to trial participants, and must also accommodate individual choices of participants with regard to the use of these modalities. We explore four potential trial designs that address these challenges, with a focus on accommodating the newest addition to the prevention package-antiretroviral-based oral pre-exposure prophylaxis (PrEP). The designs differ with respect to how individuals who take up oral PrEP at screening are handled. An All-Comers Design enrolls and randomizes all eligible individuals, a Decliners Design enrolls and randomizes only those who decline PrEP at screening, and Single and Multi-Stage Run-In Designs enroll all but randomize only those who decline PrEP or show inadequate adherence to PrEP after one or multiple run-in periods. We compare these designs with respect to required sample sizes, study duration, and resource requirements, using a simulation model that incorporates data on HIV risk and PrEP uptake and adherence among men who have sex with men (MSM) in the Americas. We advocate considering Run-In Designs for some future contexts, and identify their advantages and tradeoffs relative to the other designs. The design concepts apply beyond HIV vaccines to other prevention modalities being developed with the aim to achieve further reductions in HIV incidence.

## 1 Introduction

Dramatic advancements have been made in recent years in antiretroviral (ARV)-based prevention of HIV infection. In particular, ARV treatment of HIV-infected individuals, or Treatment-as-Prevention (TasP), has been shown to reduce the risk of HIV transmission by a dramatic 96% [8, 9], and prophylactic ARV use among HIV-uninfected individuals, or pre-exposure prophylaxis (PrEP), has been found to have high efficacy in populations that adhere to current regimens which entail daily pill taking [5, 7, 15, 17, 24, 27, 28, 38, 40]. Most recently, cabotegravir as injectable PrEP has been found to be highly effective in a trial of men who have sex with men (MSM) [32]; a trial of this same intervention in women in Africa is ongoing. Yet, HIV remains a significant public health burden with 1.7 million new infections in 2018, and 38% of HIV-infected individuals not accessing treatment [37]. Implementation of ARV-based preventive interventions has been hindered by social, behavioral, ethical, and economic factors, and uptake and sustained adherence has been variable [25, 36, 41]. Vaccines have generally been the tools used to contain and eliminate other infectious diseases and an effective HIV vaccine will ultimately be needed to bring an end to the epidemic.

The design of future vaccine efficacy trials is challenged by the rapidly evolving HIV prevention landscape [22]. Three vaccine trials underway or recently completed utilize prototypical designs that randomize HIV-negative participants to Vaccine or Placebo and follow them for HIV infection endpoints over a fixed duration of follow-up [19]. A state-of-the-art HIV prevention package that includes risk reduction counseling, free condoms, STI testing and treatment, referral for voluntary medical male circumcision, and education around and access to oral PrEP, is provided to all participants throughout the course of the trial. Going forward, this design may no longer be optimal or feasible. A specific challenge will be balancing the ethical mandate to provide participants the best standard of HIV prevention—which in turn will reduce HIV incidence among trial participants—and enabling the assessment of vaccine efficacy through an adequately powered trial. As well, designs must reflect the reality that diverse cultural, lifestyle, and biological circumstances influence individual decision-making around the use of HIV prevention strategies, and that these choices are dynamic even over the course of a trial.

This paper discusses four potential design approaches for future vaccine efficacy trials. We focus on approaches that may apply in the next few years, anticipating that oral PrEP use will increase but remain heterogeneous. In the discussion, we comment on application of the design approaches to an era in which injectable PrEP is added to the HIV prevention package. Importantly, while our focus is vaccines, the concepts and approaches also apply to evaluating other HIV prevention products under development that are alternatives to daily oral PrEP, e.g., to microbicides or on-demand products. Reflecting the consensus that has been achieved in the field in recent years, we presume that all future designs will be conducted among individuals with “unmet need”, i.e., that individuals already using and persisting in using oral PrEP at the time of screening would not be enrolled. For these individuals, a favorable risk-benefit ratio is not achieved as they have minimal HIV risk but would be subject to the potential risks that any participant of an experimental vaccine trial takes on, e.g., due to local reactogenicity and repeated blood draws. The four designs we consider take different approaches with regard to the enrollment and randomization of individuals who take up oral PrEP at screening or during the course of the trial. Our paper is structured as follows. First, we describe the four designs and the objectives they address. Second, we implement a simulation study to investigate the relative sizes and resources required for the designs, as a means of highlighting the key parameters that may influence the choice of design for a given future context. Our simulations reflect features of the HIV epidemic and current status of PrEP use among MSM in the Americas. Last, we highlight other considerations around use of these designs and discuss variations on them that deserve future exploration.

## 2 Methods

### 2.1 Study Designs

In general, we consider vaccine efficacy trial designs that randomize HIV-negative participants to Vaccine or Placebo and follow them for HIV infection endpoints over a fixed duration of follow-up. We explore four specific variations on this general design, motivated by recent consultations with stakeholders in HIV prevention. The four designs differ in how participants who take up oral PrEP are handled. Under each design, at trial screening otherwise eligible individuals are educated about oral PrEP and queried about usage and interest in it. Those who are already using oral PrEP, and who are satisfied with the product and would like to continue using it, are not eligible for participation under any design. This reflects consensus that these individuals do not have a favorable risk-benefit for inclusion in a vaccine trial. However, individuals not already using PrEP but interested in receiving it, or those who make informed decisions not to use PrEP, may be enrolled as described below. Given the challenges with adhering to oral PrEP and the many factors that affect individual usage of oral PrEP [35], these individuals may benefit from a vaccine that reduces their HIV risk. In practice, the manner in which trial sites provide PrEP to participants is expected to vary; in some instances, participants may be referred to another location where PrEP can be accessed, e.g., to a demonstration project or public health access program, and in others the study site physicians may prescribe PrEP to the participants. In either situation, the cost of PrEP is covered, including all safety monitoring and clinic visits. The provision of PrEP in the context of a vaccine trial does necessitate that all laboratory testing is done at the study site, and this is especially important for the HIV testing that is part of PrEP clinical care, given that vaccines can induce false positive test results with standard HIV diagnostics.

The first design we consider, which we call the “All-Comers Design”, enrolls and randomizes all eligible individuals to Vaccine or Placebo, without regard to their acceptance of the offer of oral PrEP at screening. Participants are followed for incident HIV infection for a fixed-24 month follow-up; this is a duration that is typical of current vaccine efficacy trial designs. Importantly, education about and access to PrEP continues throughout the duration of the trial- as part of the standard prevention package and participants may elect to take up PrEP at any time and are provided access in the same manner as those who take up PrEP at screening. The All-Comers Design serves as a useful reference when evaluating the other designs that in some fashion enrich for participants who choose not to use PrEP. This is essentially the design employed by two recent HIV vaccine efficacy trials, the recently completed HVTN 702 trial and the ongoing HVTN 705 trial.

**Fig. 1:**
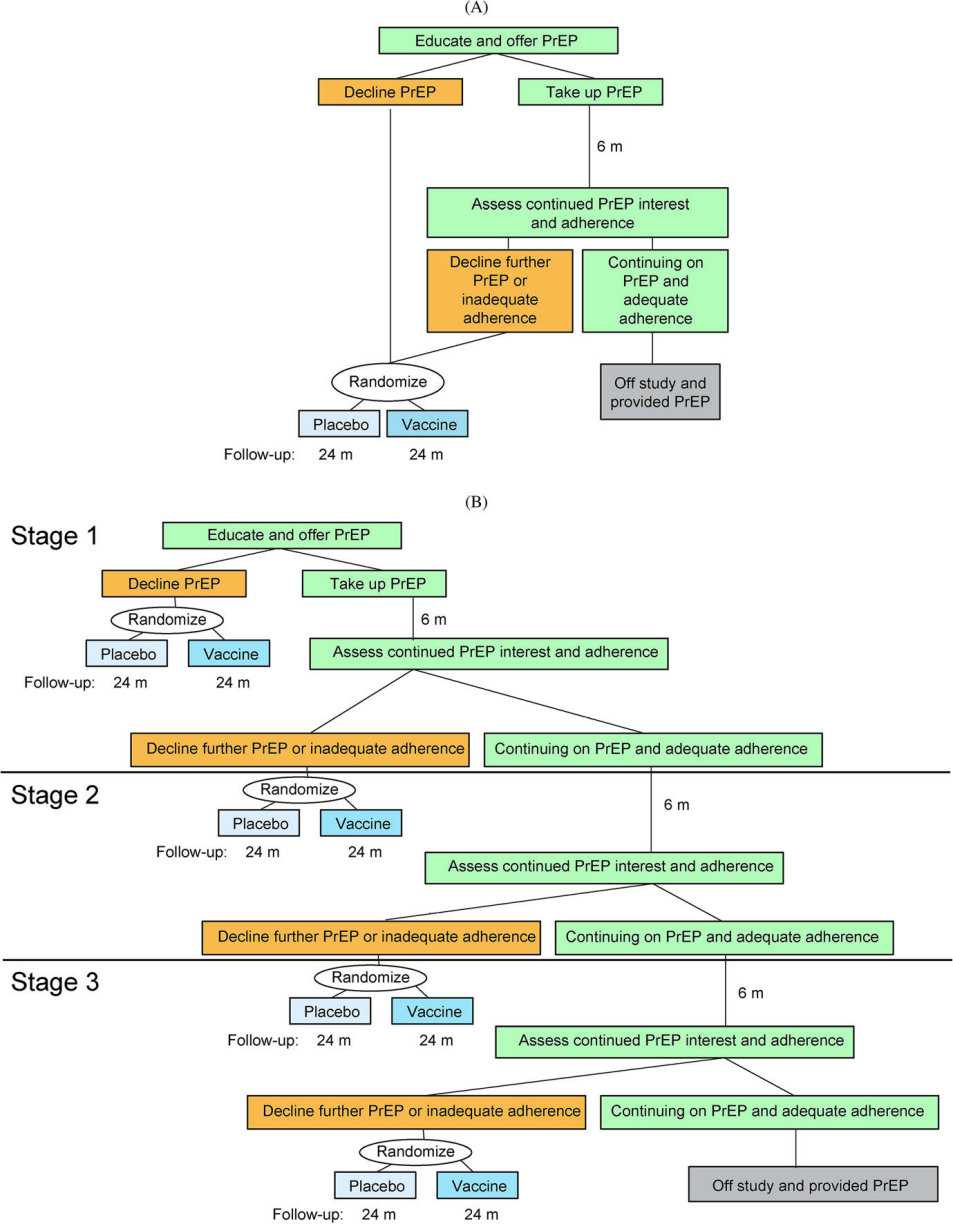
1-Stage (**a**) and 3-Stage (**b**) Run-In Designs for assessing the efficacy of a candidate HIV vaccine. Designs enroll all eligible individuals, but only randomize those who decline the offer of PrEP at screening or who decline further use of PrEP or have inadequate PrEP adherence after one or more run-in periods

The second design we consider begins with a 6-month run-in period, during which participants who take up the offer of oral PrEP at screening are enrolled and provided it. At the end of the run-in period, adherence to PrEP is assessed by measuring ARV levels in participants’ blood using standard assays [4, 42]. Only the participants without adequate adherence or who decline further use of PrEP, and who are still at risk of HIV infection, are randomized to Vaccine or Placebo and followed for HIV infection for 24 months; those who have adequate adherence and elect to continue PrEP are terminated from the study at that time point. Individuals who decline the offer of oral PrEP at screening are immediately randomized and followed for 24 months (see Fig. 1). We call this the “1-Stage Run-In Design”. The adherence threshold that is employed is a key parameter of the design, and in practice is chosen to correspond to a level of HIV incidence at which there is a favorable benefit/risk of randomization to Vaccine. Importantly, under this design all participants, including randomized participants, continue to have education around and access to oral PrEP throughout the duration of follow-up. The 1-Stage Run-In Design is expected to be more efficient than the All-Comers Design, by virtue of the enrichment for individuals who choose not to use PrEP or who are not adherent to it after the run-in period.

The third design is an extension of the previous design (Fig. 1). Under the “3-Stage Run-In Design”, three different run-in periods are used. At the end of each period, participants’ PrEP adherence is assessed through measuring blood ARV levels, and individuals without adequate adherence or who decline further use of PrEP and who are still at risk of HIV infection are randomized to Vaccine or Placebo and followed for 24 months. As under the 1-Stage Run-In Design, individuals who decline PrEP at screening are enrolled and immediately randomized and followed. Individuals who continue to be interested in using PrEP and who have adequate drug levels at the end of the third run-in period are terminated from the study at that time point. All participants, including those randomized, continue to have ongoing education around and access to oral PrEP throughout the duration of follow-up. While the design we consider employs three run-in periods, the concept is general and the number of run-in periods is a design parameter. The 3-Stage Run-In Design may have improved efficiency relative to the 1-Stage Run-In, if some individuals take more than one run-in period to determine whether oral PrEP works for them.

The fourth and last design we consider is called the “Decliners Design”. At screening, individuals who express an interest in initiating PrEP are provided it but are not enrolled. Only the individuals who decline the offer of PrEP at screening are enrolled and randomized. This design is the most aggressive of the four considered in the manner in which it enriches for participants who choose not to use oral PrEP. Again, however, ongoing education around and access to oral PrEP is provided and participants may choose to take up PrEP at any time. The Decliners Design concept is being employed in the ongoing HVTN 706 HIV vaccine efficacy trial.

An important attribute of all designs is that individuals who are not enrolled due to interest in PrEP (under the Decliners Design) or who are enrolled but not randomized (under the 1- or 3-Stage Run-In Designs) continue to have PrEP provided and covered by the study for 24 months, if they wish to continue using it. This is for the protection of these individuals, and it also removes a potential incentive for individuals to enroll or change their responses or behavior, simply to access PrEP. This strategy is being pursued for the HVTN 706 trial.

Table 1 outlines the primary efficacy and key PrEP-related secondary objectives that can be assessed with each design. These are fundamental for gauging the relative merits of the designs. Notably, the population randomized to Vaccine or Placebo differs across the designs, and therefore the vaccine efficacy (VE) parameter that is assessed differs. While the All-Comers Design evaluates VE for the most expansive population and the Decliners Design the most restrictive population, the Run-In Designs evaluate VE for intermediate-sized populations that are more difficult to characterize as they depend on outcomes of run-in periods.

Current vaccine efficacy trial designs prospectively collect and store blood specimens for all trial participants. At the end of the trial these specimens may be assayed for HIV-infected cases and frequency-matched controls to permit a variety of secondary analyses, including to assess VE among individuals not on PrEP at HIV acquisition (who either declined PrEP or took up PrEP post-enrollment were not adherent). As shown in Table 1, all designs considered here could have such specimen collection and assaying performed to assess this secondary objective.

Under a variation on the Run-In Designs, individuals who remain interested and adherent to PrEP after each run-in period continue to be followed for incident HIV infection. This variation allows two additional secondary objectives to be addressed (Table 1). Specifically, HIV incidence among PrEP users can be evaluated and used to evaluate PrEP effectiveness, by comparing HIV incidence among PrEP users vs. those randomized to Placebo, and also to compare Vaccine and PrEP effectiveness, by comparing HIV incidence among PrEP users vs. those randomized to Vaccine. To address these objectives, however, statistical methods would need to control for the many possible differences in risk between individuals who do and do not choose to use PrEP.

For the purposes of comparing the operating characteristics of the designs in the next section, we focus on their capacity to address the primary vaccine efficacy objectives. In the discussion, we comment on relative power of the designs to address Secondary Objective 1.

### 2.2 Simulations to Compare Study Designs

We use a model that links PrEP, vaccine, and HIV infection status to simulate data for an MSM population in the Americas. The simulations are used to compare the design attributes for a few specific scenarios of interest, and to identify the parameters that may influence the choice of design more generally.

#### 2.2.1 Simulation Model

To capture the heterogeneity in HIV risk in efficacy trial populations-attributable to demographic and risk behavior characteristics—we assume that there are three latent HIV risk groups in the absence of PrEP or Vaccine, denoted by *W* ∈ {1, 2, 3} and called low, average, and high risk. See the full set of model parameters in Table 2. We assume there are equal proportions of individuals in each risk group. The time of HIV infection, *Y*, is assumed to follow an exponential distribution with a marginal annual incidence of 3 per 100 person-years (*λ*_0_), and the hazard ratios for the three risk groups are *HR*_*W*_ = (0.5, 1.0, 2.0) which results in incidence rates of 1.5, 3 and 6 per 100 person-years, respectively. The marginal incidence of 3 per 100 person-years is consistent with rates seen in recent efficacy trials in the MSM population in the Americas [21]. Sensitivity analyses described in Online Resources show the results for 4% marginal incidence.

**Fig. 2:**
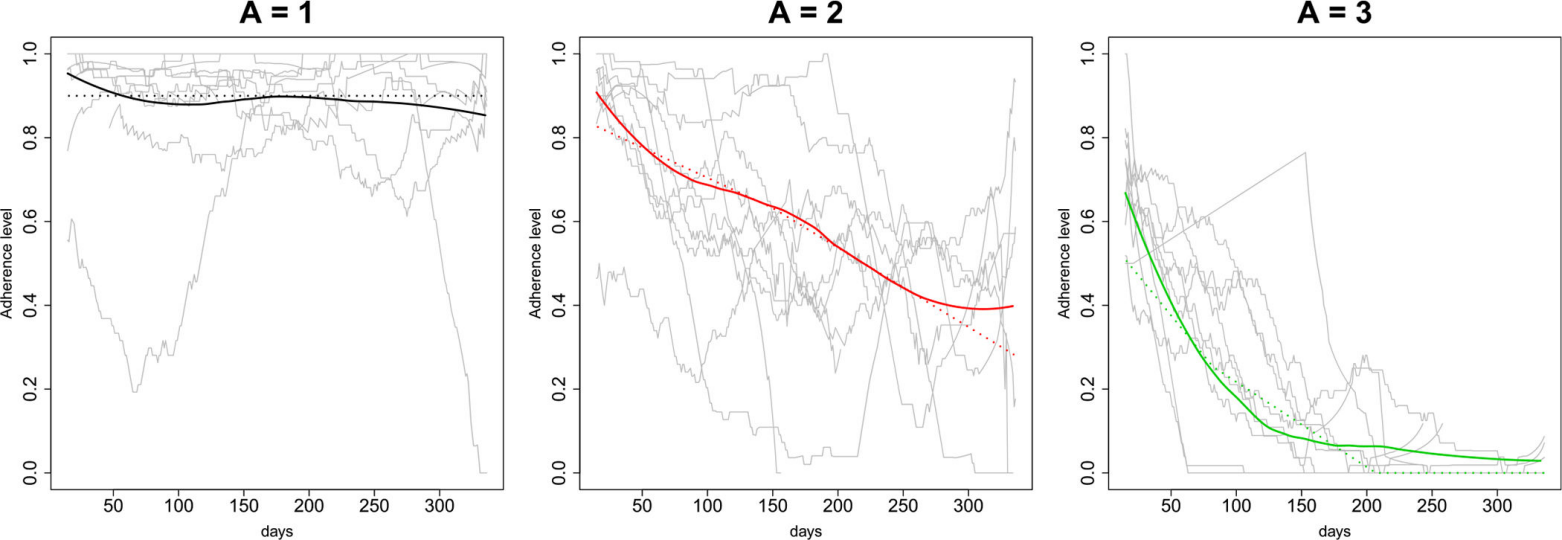
Adherence to oral PrEP in HPTN 069 based on daily Wisepill™ electronic device monitoring. Based on these data, participants are determined to fall in to one of three PrEP adherence categories. Each panel corresponds to a specific adherence category and shows the adherence trajectory for a random sample of 10 participants (gray), along with the empirical mean adherence trajectory (solid color) and an estimated mean adherence trajectory based on the fitted linear change-point model (dashed)

Individuals are assumed to belong to one of three latent PrEP adherence groups, denoted by *A* = 1, 2, 3 (see Fig. 2). The groups are based on data from HPTN 069 [18], a phase II PrEP safety and tolerability study in MSM and at-risk women in the US and Puerto Rico. In this study, Wisepill™ electronic device monitoring was used to measure participants’ daily pill-taking. Linear change-point models fitted to the HPTN 069 data (*n* = 406 MSM and *n* = 188 women; *n* = 182 subjects with dense Wisepill™ data) [43] identified three adherence groups, corresponding to consistently high adherence, slowly declining adherence, and rapidly declining adherence as measured by the fraction of pills taken per week, with group membership probabilities *P*(*A* = *a*) ∈ {.4, .3, .3}. We use these previously published latent PrEP adherence groups, and assume that once an individual in group *A* = *a* takes up the offer of PrEP, the latent individual-level adherence trajectory, defined in terms of the fraction of prescribed pills taken and denoted by *A*(*t*) = *μ*(*t*) + *σ*(*t*), is assumed to follow the time-varying linear change-point adherence model with mean process *μ*_*a*_(*t*) = *μ*_0*a*_ + *β*_1*a*_(*t* ∧ *t*_0*a*_) + *β*_2*a*_*tI*(*t* > *t*_0*a*_) where *t*_0*a*_ is the change-point, and additive random noise process $\sigma_{a}(t)\sim N\left(0,\sigma_{a}^{2}\right)$. To account for additional measurement error in the adherence measurements we assume that the observed adherence trajectory is *A*^*obs*^(*t*) = *μ*(*t*) + σ*(*t*), where $\sigma_{a}^{*}(t)\sim N\left(0,4\cdot \sigma_{a}^{2}\right)$. See Online Resources for the parameter values. This model allows for variability among individuals within an adherence group, but importantly it assumes that expected adherence declines monotonically over time. The HPTN 069 data form a useful basis for the adherence model because they are rich in time and therefore permit simulating daily individual-level adherence measurements. Qualitatively, the HPTN 069-based adherence model is consistent with recent demonstration project data showing modest uptake and persistence of PrEP use among MSM in the Americas [11, 20, 33, 34].

Given that many PrEP efficacy trials have found that factors that associate with higher HIV risk also tend to predict lower adherence to oral PrEP [2, 12, 27], our model allows for potentially correlated latent adherence and risk groups. Specifically, we assume the marginal responses of *A* and *W* are derived from probit-transformed latent continuous random variables that are bivariate normal with correlation *ρ*_*AW*_; see [39] for details. Qualitatively, positive *ρ*_*AW*_ is consistent with low-risk individuals tending to be more adherent, while negative *ρ*_*AW*_ indicates that high-risk individuals tend to be more adherent.

Reflecting the current context in which PrEP uptake is heterogeneous, our model allows for a fraction of participants to decline the offer of PrEP at screening, and for some of these initial decliners to take up PrEP at some time point post-screening. Specifically, let $t_{i}^{\text{uptake}}$ be the PrEP uptake time for subject *i*. Individuals who take up the offer of PrEP at screening have *t*^uptake^ = 0. Individuals who decline PrEP at screening have *t*^uptake^ > 0; some will never take up PrEP during the maximum 42-month follow-up across designs, i.e., *t*^uptake^ > *T* where *T* is the maximum follow-up time, and others will take up PrEP at certain follow-up visit after initial enrollment (0 < *t*^uptake^ < *T*). We assume a fixed value for *P*(*t*^uptake^ = 0) and that post-screening PrEP uptake is uniformly distributed across 3-monthly follow-up visits until truncation at time *T*. Therefore the distribution of min{0, *t*^uptake^} is a zero-and-*T*-inflated uniform distribution at 3-month follow-up intervals between first (*t*^uptake^ = 3 months) and last (*t*^uptake^ = *T*) visits, where *T* = 42 months. Based on data from the ongoing HVTN 704 monoclonal antibody prevention trial in MSM in which oral PrEP is offered to participants and uptake is approximately 25% (Peter Gilbert, personal communication), but anticipating increasing uptake in coming years, we set *P*(*t*^uptake^ = 0) = 0.5, *P*(0 < *t*^uptake^ < *T*) = 0.3, and *P*(*t*^uptake^ > *T*) = 0.2. At $t_{i}^{\text{uptake}}$, subject *i*’s adherence trajectory is assumed to follow from the linear change-point model for adherence category *A*_*i*_. We assume that *A*_*i*_(*t*) = 0 for $t<t_{i}^{\text{uptake}}$, i.e., that participants do not procure PrEP outside the study. If $t_{i}^{\text{uptake}}>T$ we set *A*_*i*_(*t*) = 0 for all *t*. Importantly, this model assumes that PrEP uptake is independent of latent adherence and HIV risk categories. Sensitivity analyses shown in Online Resources consider lower rates of PrEP uptake.

Data from PrEP efficacy trials and studies with directly observed dosing [3, 16] form the basis for our model linking time-varying adherence *A*(*t*) with HIV outcomes. We assume the hazard ratio associated with PrEP follows

(1) $$HR_{PrEP}(A(t))=e^{-\theta_{1}A{\left(t\right)}^{\theta_{2}}}.$$

**Fig. 3:**
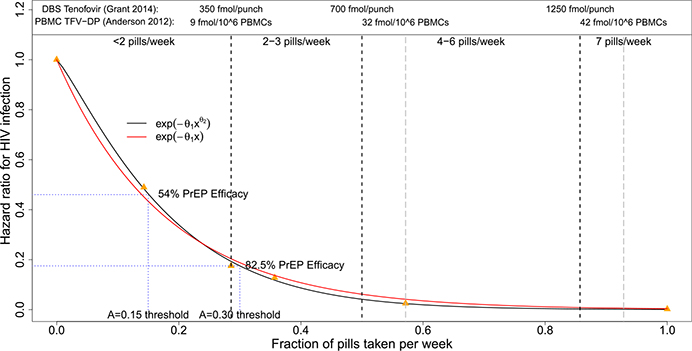
Hazard ratio for oral PrEP as a function of adherence, as measured by fraction of pills taken per week. The orange triangles are estimates based on published work by [3, 16] and the red and black curves are estimates of the association based on Model 1 that assumes *θ*_2_ = 1 (red curve) or that estimates *θ*_2_ using least-squares estimation (black curve). Corresponding drug concentration levels in dry blood spots (DBS) [16] or peripheral blood mononuclear cells (PBMCs) [3] are shown at the top. The 0.15 adherence threshold that prompts randomization in Run-In designs corresponds to an estimated PrEP hazard ratio of 0.46

An exponential model with *θ*_2_ fixed to 1 was used in [3] to estimate the association between adherence, as measured by tenofovir concentration in peripheral blood mononuclear cells (PBMCs), and HIV risk among MSM on PrEP, based on data from the iPrEX trial [15]. Figure 3 shows point estimates of adherence and PrEP hazard ratios from [3, 16], based on calculations that convert drug concentration levels in PBMCs or dried blood spots to the fraction of pills taken using the published relationship between dosing and drug concentration [3, 6], and that assume that risk among placebo recipients does not vary with adherence to daily pill-taking. We estimated the parameters *θ*_1_ and *θ*_2_ by fitting Model 1 to the points in Fig. 3 using least-squares. This model motivated the choice of PrEP adherence threshold below which the Run-In Designs randomize a participant who takes up PrEP. The threshold *A*_0_ = 0.15 corresponds to an estimated PrEP efficacy of 54%; this is a level of PrEP efficacy below which it may be deemed ethical to randomize an individual to ascertain whether the vaccine can reduce HIV risk even further.

Finally, we assume that participants are randomized to Vaccine (*Z* = 1) or Placebo (*Z* = 0) with equal probability. Vaccine efficacy is measured by the multiplicative reduction in HIV risk due to assignment to vaccine, *VE* = 1 − *HR*_*V*_, and is assumed constant over 24 months follow-up. The vaccine is also assumed not to interact with HIV risk or usage of oral PrEP. Given (*W*, *A*, *Z*), *λ*(*t*|*W*, *A*(*t*), *Z*) = *λ*_0_ · *HR*_*W*_ · *HR*_*PrEP*_(*A*(*t*)) · (1 − *VE*)^*Z*^ is the instantaneous hazard of HIV infection. The cumulative probability distribution function of *Y* is $F_{Y}(t|W,A,Z)=1-e^{-\int_{0}^{t}\lambda (s|W,A(s),Z)ds}$. We assume an exponentially distributed non-informative censoring time, *C* ~ *Exp*(*λ*_*c*_), independent of (*W*, *A*, *Z*, *Y*), and a 10% annual censoring rate. Table 2 describes the full set of model parameters and assumed values.

To capture the resources required for the designs, we describe the number of participants who must be screened and enrolled to achieve adequate power and discuss the implications with respect to accrual time. We also characterize the resources required for PrEP provision and adherence monitoring. For the purposes of this work, we make the simplifying assumption that the time of screening is the same as the time of enrollment. In practice, there may be a small gap of several days between the two time points.

#### 2.2.2 Simulation Algorithm

We describe the steps in simulating the data for the All-Comers and 1-Stage Run-In Designs, and briefly highlight the major differences in approaches to the 3-Stage Run-In and Decliners Designs below.

For the All-Comers Design, we begin by simulating each individual’s latent HIV risk and adherence group membership, *W*_*i*_ ~ *p*_*W*_(*w*) and PrEP adherence *A*_*i*_ ~ *p*_*A*_(*a*) for *i* = 1, … , *n*, based on the bivariate normal latent variables with correlation *ρ*_*AW*_. Next, we simulate each individual’s PrEP uptake time, $t_{i}^{\text{uptake}}$. The individual-level PrEP adherence trajectory is simulated from the time of PrEP uptake, according to the linear change-point model for group *A*_*i*_. Given a random treatment assignment *Z*_*i*_ and a censoring time *C*_*i*_ we next simulate the HIV infection time *Y*_*i*_ with the cumulative intensity process $\Lambda_{i}(t)=\int_{0}^{t}\lambda_{0}\cdot HR_{W_{i}}\cdot HR_{PrEP}\left(A_{i}(s)\right)ds$. Let $F_{i}(t)=1-e^{-\Lambda_{i}(t)}$. We use inverse transform sampling to simulate $Y_{i}=F_{i}^{-1}(U)$ where *U* ~ *Unif*[0, 1] and then calculate the observed event time $Y_{i}^{obs}=min\left(Y_{i},C_{i},24\;\text{months}\right)$ and censoring indicator $\delta_{i}=1-I\left(Y_{i}=Y_{i}^{obs}\right)$. The Decliners Design is simulated similarly but randomization only occurs for those with $t_{i}^{\text{uptake}}>0$.

For the 1-Stage Run-In Design, *W*_*i*_, *A*_*i*_, $t_{i}^{\text{uptake}}$, and *A*_*i*_(*t*) are simulated as above. For individuals with $t_{i}^{\text{uptake}}=0$, data for the 6-month run-in are generated as follows. The infection time during 1-Stage Run-In, $Y_{i}^{1}$, is generated using the intensity process $\lambda_{i}^{1}(t)=\lambda_{0}\cdot HR_{W_{i}}\cdot HR_{PrEP}\left(A_{i}(t)\right)$. The censoring time, $C_{i}^{1}$ follows an exponential distribution with intensity *λ*_*C*_. The measured adherence level at the end of the 6-month run-in period is denoted by $A_{i}^{obs}(t=6\;\text{month})$. Randomization occurs for those with $t_{i}^{\text{uptake}}=0$, $A_{i}^{obs}(6\;\text{month})<A_{0}$ and $min\left(Y_{i}^{1},C_{i}^{1}\right)\geq 6\;\text{months}$. Let $\delta_{i,1}^{V_{trial}}=I\left[min\left(Y_{i}^{1},C_{i}^{1}\right)\geq 6\;\text{months}\right]*I\left[A_{i}^{obs}(6\;\text{month})<A_{0}\right]*I\left[t_{i}^{\text{uptake}}=0\right]$ be an indicator of satisfying these conditions. We generate *Z*_*i*_ for each individual with $\delta_{i,1}^{V_{trial}}=1$. We simulate the post-randomization outcome $Y_{i}^{2}$ with intensity process $\lambda_{i}^{2}(t)=\lambda_{0}\cdot HR_{W_{i}}\cdot HR_{PrEP}\left(A_{i}(t+6\;\text{month})\right)\cdot {\left(1-VE\right)}^{Z_{i}}$ similar to the generation process of $Y_{i}^{1}$, and $C_{i}^{2}$ as exponential with rate *λ*_*C*_. The observed post-randomization event time is $Y_{i}^{2,obs}=min\left(Y_{i}^{2},C_{i}^{2},24\;\text{months}\right)$ and the censoring indicator is $\delta_{i}^{2}=1-I\left(Y_{i}^{2}=Y_{i}^{2,obs}\right)$. Individuals with $t_{i}^{\text{uptake}}>0$ are randomized at enrollment and have outcomes generated as under the All-Comers Design.

Under the 3-Stage Run-In Design, subject *i* is randomized after the *k*th run-in stage if $\delta_{i,k}^{V_{trial}}\prod_{l=1}^{k-1}\left(1-\delta_{i,l}^{V_{trial}}\right)=1$, where $\delta_{i,k}^{V_{trial}}=I\left[min\left(Y_{i}^{1},C_{i}^{1}\right)\geq 6*k\;\text{month}\right]*I\left[A_{i}^{obs}(6*k\;\text{months})<A_{0}\right]*I\left[t_{i}^{\text{uptake}}=0\right]$. Post-randomization event times $\left(Y_{i}^{k},\;\text{s},k=2,3,4\right)$ are generated as under the 1-Stage Run-In Design, although the subjects’ time-varying hazard function will begin at *t* = 6 * *k* months instead of *t* = 0, since the 24 months follow-up period after run-in randomization excludes previous run-in periods.

##### 2.2.2.1 Power Comparison

We compare the power of the designs to reject H_0_: VE ≤ 25% under the alternative H_*a*_: VE = 50% in the randomized and modified intent-to-treat (MITT) population, defined as the set of randomized participants who are retrospectively determined to have been HIV-uninfected at the time of randomization. The choice of null and alternative hypotheses is consistent with recent vaccine efficacy trial designs [13], and is based on consideration of properties of a minimally useful vaccine and informed by the anticipated level of efficacy of current vaccine regimens.

An 0.05-level 2-sided log-rank test is used to evaluate VE in the randomized MITT population. For the 1-Stage and 3-Stage Run-In Designs, the test is stratified on the stage of randomization for improved power.

Note that even though the designs enroll and randomize different populations (see Table 1), under our assumption that the vaccine does not interact with baseline risk, PrEP uptake, or PrEP adherence the true VE parameter is the same across all populations. Therefore all four designs produce unbiased VE estimates.

We calculate empirical power by simulating designs for a grid of possible total sample sizes, calculating the probability of *H*_0_ rejection under *H*_*a*_ for each sample size based on 1000 simulations, and plotting the results to find the number needed to randomize to achieve 90% power.

## 3 Results

### 3.1 Required Sample Sizes

Table 3 shows the fraction of participants randomized at enrollment and after each run-in period for each of the designs. While individuals who decline PrEP at screening are immediately randomized under all designs, those who take up PrEP at screening follow different paths depending on the design. For the All-Comers Design, those who take up PrEP at screening are also immediately randomized, while for the Decliners Design those who take up PrEP at screening are not enrolled (or randomized). For the 1- and 3-Stage Run-In Designs, the time point at which participants are randomized depends on what PrEP adherence category they fall in to. Those who fall in to the consistently high adherence category (*A* = 1) never have adherence levels that fall below the adherence threshold of 15% of pills per week, and thus are never randomized under the Run-In Designs. Those in slowly declining adherence category (*A* = 2) tend to be randomized after two or three run-in periods; a small fraction have low enough adherence to be randomized after one run-in period. Those with rapidly declining adherence (*A* = 3) quickly achieve low adherence levels and are randomized after one run-in period for both Run-In Designs.

Table 4 compares the numbers of participants involved in each of the trial designs, where we distinguish between the number of individuals who are offered PrEP at screening, the number who are enrolled, and the number who are randomized to Vaccine or Placebo and used to evaluate vaccine efficacy. Results are shown for three different values of *ρ*_*AW*_, the correlation between baseline risk and PrEP adherence.

For the All-Comers Design, the numbers offered PrEP, enrolled, and randomized are the same- and are large by virtue of the lower placebo-group incidence due to PrEP.

The Decliners Design, which enrolls and randomizes only those who decline PrEP at screening, requires considerably fewer participants, 7000 vs. 9000, or a 22% reduction in sample size under *ρ*_*AW*_ = 0. However, under our assumptions, in order to enroll these 7000 participants, PrEP is provided for 24 months to an additional 7000 individuals who are not enrolled and do not contribute to meeting the scientific objectives of the trial. This is an important consideration in terms of resources.

The 1-Stage Run-In Design requires slightly fewer participants to achieve 90% power. With *ρ*_*AW*_ = 0, 6762 are required vs. 7000 for the Decliners Design, and thus the 1-Stage Run-In achieves a 25% reduction in sample size relative to the All-Comers Design. Most of those randomized are randomized at enrollment (83%) as opposed to after the single run-in period. The slight gain in power relative to the Decliners Design is due to the enrollment and randomization of individuals who take up PrEP at screening but who fall into the low adherence category (see Table 3), and who therefore have an HIV incidence that is minimally reduced by PrEP.

An important cost consideration of the 1-Stage Run-In Design is that many participants enrolled are provided PrEP for the run-in period and have adherence assessed at its end, but are not randomized (43% under *ρ*_*AW*_ = 0); these participants do not contribute to assessing vaccine efficacy. The design accrues the cost of enrolling and following participants for the run-in period for the purposes of adherence assessment, and this needs to be factored in to cost assessments. As well, the 1-Stage Run-In Design is longer in duration than the All-Comer or Decliners Designs, with 30 months maximum follow-up as compared to 24 months follow-up per participant under the other designs.

Interestingly, under our model the 3-Stage Run-In Design is less powerful than the 1-Stage Run-In Design. With *ρ*_*AW*_ = 0, a total of 7265 individuals must be randomized to achieve 90% power vs. 6762 for the 1-Stage Run-In Design. The reason for the lower trial power is that the only difference between the designs is the randomization of a relatively small fraction of individuals who take up PrEP at screening and who fall into the intermediate adherence category (see Table 3), and these individuals are randomized at the expense of those who decline PrEP or who have low PrEP adherence and who have higher HIV incidence. Note that the vast majority of those randomized under the 3-Stage Design are randomized at enrollment (63%) or after the first run-in (14%). Also observe that the 3-Stage Run-In Design is considerably longer in duration than the other designs, with a maximum follow-up time of 42 months as compared to 24 months for the All-Comers and Decliners Designs and 30 months for the 1-Stage Run-In Design. However, in contrast to the 1-Stage Run-In Design, most (76% under *ρ*_*AW*_ =0) of those enrolled are randomized and contribute to evaluating vaccine efficacy.

Under the likely scenario in which higher risk subgroups are likely to be less adherent (*ρ*_*AW*_ = 0.5), the results are similar, with the 1-Stage Run-In Design achieving the smallest number of randomized participants (75% the size of the All-Comers Design). All four designs require fewer participants to have 90% power, since PrEP has a smaller effect on HIV risk. When the opposite is true and lower risk subgroups are likely to be less adherent (*ρ*_*AW*_ = −0.5), all four designs require more participants, and the designs that enrich for individuals who choose not to use PrEP are less powerful relative to the All-Comers Design because under this scenario enriching for poor adherence is akin to enriching for lower risk.

**Fig. 4:**
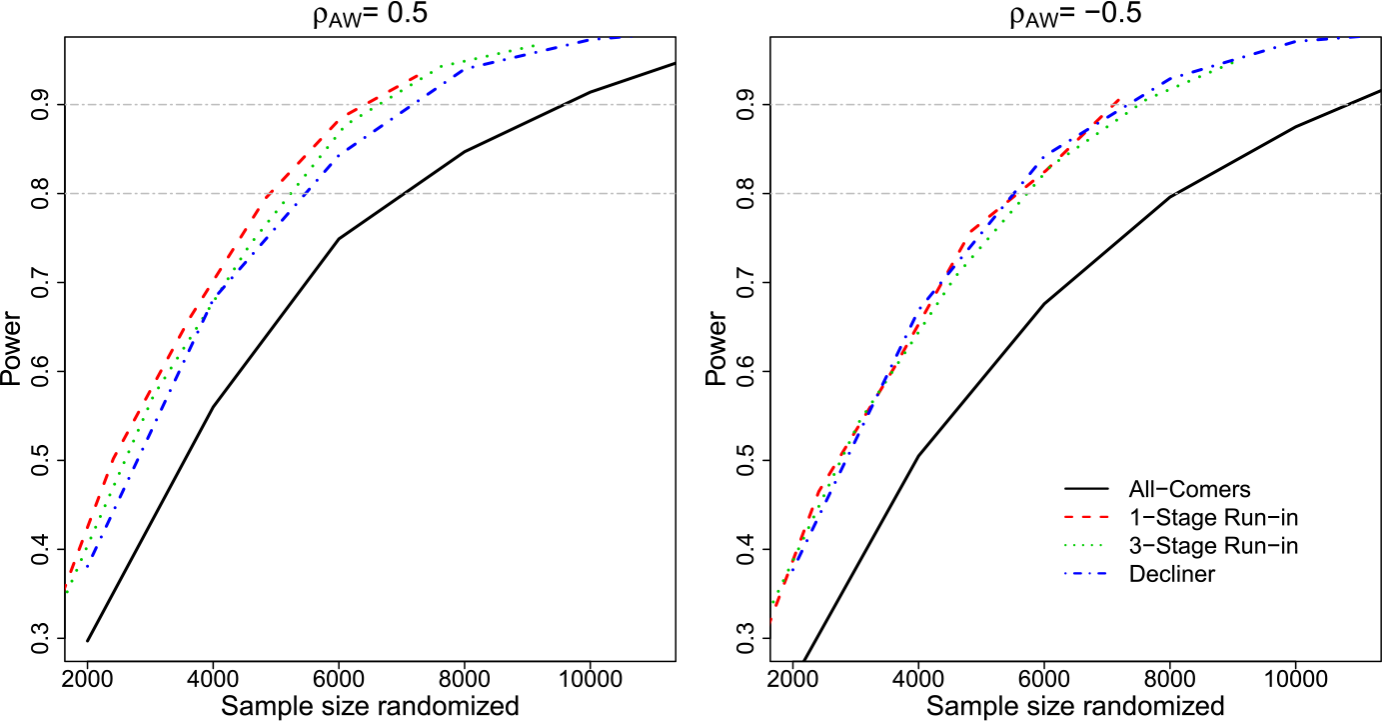
Empirical power to detect *H*_*a*_ : *VE* = 50% versus *H*_0_ : *VE* = 25% as a function of the total number of randomized participants for each proposed study design. Power is based on 1000 simulations and a 2-sided 0.05-level log-rank test (stage-stratified for Run-In Designs), and is shown for a scenario in which low-risk individuals tend to be more adherent to PrEP (*ρ*_*AW*_ = 0.5, left) and a scenario in which high-risk individuals tend to be more adherent (*ρ*_*AW*_ = −0.5, right)

The impact of the enrichment for individuals who choose not to use PrEP is visually apparent in Fig. 4, which compares the power of the designs as a function of the number of participants randomized to Vaccine or Placebo (*N*_*R*_). The All-Comers Design, which enrolls without regard to PrEP uptake and adherence, has the lowest power for a fixed *N*_*R*_ among the designs. The 1-Stage Run-In Design has the highest power, by virtue of its randomization of individuals who decline PrEP or are not adherent after 6 months; with 5000 participants randomized when *ρ*_*AW*_ = 0.5, the 1-Stage Run-In Design has 80% power as compared to 66% for the All-Comers Design. The 3-Stage Run-In Design has slightly lower power at 77% and the Decliners Design has 75% power.

When *ρ*_*AW*_ = −0.5, larger sample sizes are required to achieve the same power for all four proposed designs. Interestingly, in this scenario the Run-In and Decliner Designs are quite similar to each other, and the 1-Stage Run-In Design has less advantage over the 3-Stage Run-In Design.

### 3.2 Study Resource Requirements

**Fig. 5:**
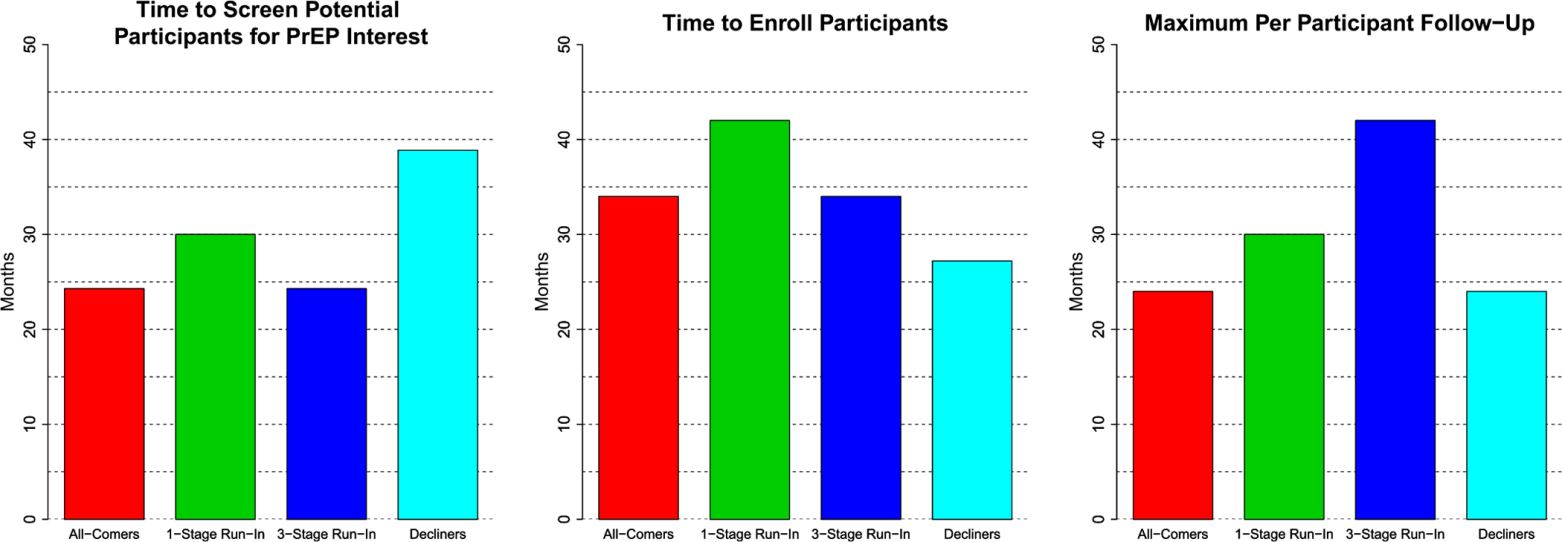
Duration of time needed to screen individuals for interest in PrEP, enroll participants, and maximum follow-up time per participant, for each proposed study design when *ρ*_AW_ = 0.5. While calculations assume 350 individuals can be screened per month and 250 enrolled per month, the relative durations are invariant to these rates

Figure 5 summarizes the expected duration of the PrEP screening, enrollment, and follow-up periods for the four designs. To enable their comparison, we assume that 350 individuals can be screened for interest in PrEP per month-with PrEP access provided if interested- and that 250 trial participants can be enrolled per month. However, the relative screening and enrollment periods of the designs are invariant to these assumed rates. Typically, screening and enrollment periods would occur in parallel, but examining them separately shows where the time resources are invested for each of the designs. We see that, by virtue of the Decliners Design’s need to screen large numbers of individuals to enroll only those who decline PrEP at screening, this design has the longest screening period, but also the shortest enrollment and per-participant follow-up periods; the Run-In Designs require shorter screening but longer enrollment and follow-up times.

**Fig. 6:**
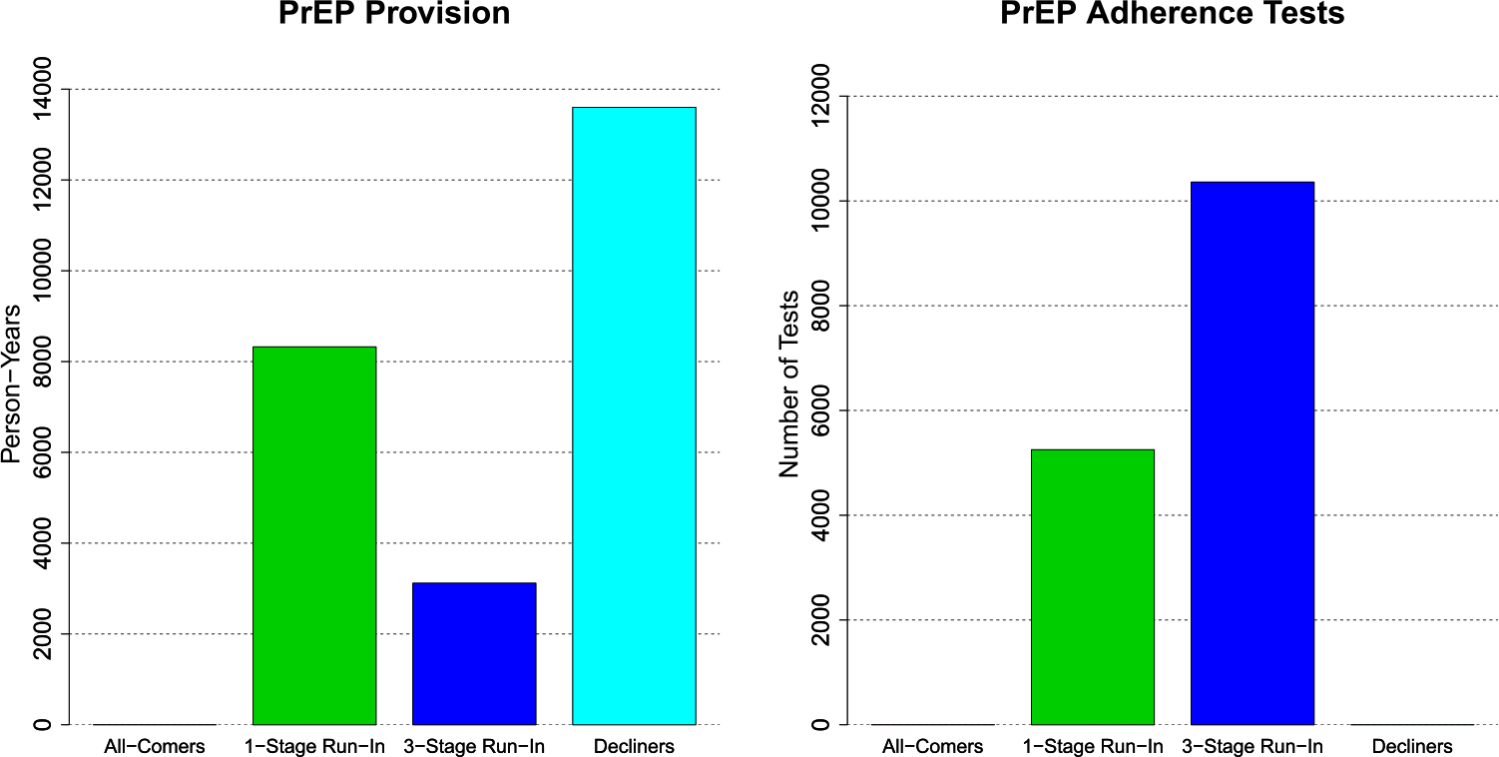
Person-years of off-study PrEP provision and number of PrEP adherence tests required for each of the proposed study designs when *ρ*_AW_ = 0.5. Off-study PrEP includes 24 months of PrEP for individuals who are screened for PrEP interest but not enrolled, and 24 months PrEP provision, starting at enrollment, for participants continuing PrEP after one or multiple run-in periods who are not randomized. PrEP adherence testing is performed at the end of each run-in period for the Run-In Designs

Figure 6 compares the designs with respect to the person-years of off-study PrEP provision. Off-study PrEP includes 24 months of PrEP for individuals who are screened but not enrolled, and 24 months of PrEP for participants continuing PrEP after one or more run-in periods, who are not randomized and therefore do not contribute to meeting primary study objectives. The designs are also compared with respect to required number of PrEP adherence tests. The figure emphasizes that the Run-In and Decliners Designs involve providing considerable person-years of off-study PrEP. The Run-In Designs also require PrEP adherence testing that the other designs do not. These factors must be weighed against any decrease in sample size.

### 3.3 Influence of Simulation Model Parameters

Parallel results for additional parameter settings are contained in Online Resources. The relative power of the designs is found to be similar in general for higher marginal HIV incidence. When the rate of PrEP uptake at screening is assumed to be lower (25% vs. 50%) and the fraction who never take up PrEP is higher (30% vs. 20%), the All-Comers Design is seen to be more powerful but still not competitive against the Run-In or Decliners Designs. When the PrEP adherence threshold is increased from *A*_0_ = 0.15 to *A*_0_ = 0.3, corresponding to 82.5% PrEP efficacy (see Fig. 3), the Run-In Designs have slightly improved power and require smaller *N*_*R*_ relative to the All-Comers Design.

A parameter with particular influence is the patterns of PrEP adherence. Under our simulation model, motivated by the HPTN 069 data, the majority of individuals have stable adherence or rapidly declining adherence. In populations with sizeable subgroups with slowly declining adherence, the 3-Stage Run-In Design is expected to have improved power.

Another parameter that is influential in general is the length of the run-in periods, which we took to be 6 months long. As shown in the Online Resources, when a shorter 3-month run-in period is used, the 3-Stage Run-In Design has higher power (requires fewer randomized participants) compared to all other designs as long as *ρ* = 0 or 0.5. This is due to the less frequent randomization of individuals in the intermediate (*A* = 2) adherence category.

## 4 Discussion

This paper compared four potential designs for future HIV vaccine efficacy trials that take into account what is anticipated to be increasing but variable use of oral PrEP in at-risk populations- both within and among individuals. While recent HIV vaccine efficacy trials (HVTN 702 and 705) utilize an “All-Comers” approach that enrolls participants without regard to PrEP uptake, and a trial just underway (HVTN 706) is using a “Decliners” approach that only enrolls those who decline oral PrEP at screening, we found that Run-In Designs that provide interested individuals with PrEP for fixed durations of time, assess interest and adherence at the end of run-in periods, and randomize those not interested in continuing PrEP or not adherent to it, can require smaller numbers of randomized participants. However, this sample size advantage must be traded off against longer duration of follow-up, the additional cost of measuring adherence to PrEP, and the cost of providing PrEP to individuals during run-in, before randomization.

An important attribute of the various designs is the relative interpretability of their vaccine efficacy parameter estimates. While the efficacy estimates from the All-Comers and Decliners Designs are simple to interpret, those from the Run-In Designs are not, since the populations they apply to are defined based on outcomes measured after run-in(s). Thus, another limitation of the Run-In Designs is parameter interpretability, and this must be weighed against the potentially reduced sample size.

There are additional challenges with implementing the proposed designs that require separate investigation. How will trial feasibility be assessed, in the face of uncertain PrEP uptake and adherence in advance of the trial? Current efficacy trials utilize operational futility monitoring plans that assess HIV incidence in the treatment-arm-pooled trial population, to enable design modification or termination for operational futility if HIV incidence is lower than anticipated. Will such monitoring among randomized participants suffice, or will additional procedures be needed? As well, will there be licensure implications if vaccine efficacy is evaluated using a design that in some way enriches for individuals not using PrEP? In particular, will regulators be concerned with the generalizability of the trial results, and the safety and efficacy of the vaccine in populations with higher rates of PrEP use- and how will these concerns be addressed? Also challenging, how will community and site investigators be engaged meaningfully to permit such designs, which will require careful communication regarding the scientific and ethical rationale for the designs and the procedures in place to protect participant safety? The designs discussed will clearly not be possible without robust support from these key stakeholders. Furthermore, how will participant informed consent be procured? Participants will need to demonstrate not only an understanding of the vaccine and its potential risks and benefits but also of PrEP and the trial’s approach to it, and to authentically choose whether and when to use PrEP [23]. Finally, will retention of trial participants be more challenging with these designs? Given that some of the barriers to sustained adherence to PrEP may also be barriers to attending clinic visits and adhering to other clinical procedures, there is a potential for participants who choose not to continue on PrEP to be harder to retain on-study. How will the designs anticipate and tackle this issue? The challenges summarized here are considerable.

In exploring the proposed designs, our simulation study did not endeavor to exhaustively examine the potential scenarios in which one design may be preferred over another. Instead, our goal was to illustrate and compare the designs in a handful of reasonable scenarios, and to use the simulations to identify parameters that are especially influential in terms of design optimality. Online Table 10 lists the key attributes of the target population, the study design, and the clinical context, and highlights those that will have major impact on the relative power of the designs we considered. These include the patterns of PrEP adherence, uptake, and the efficacy of PrEP in the target population; and the length of the run-in period and the PrEP adherence randomization threshold which are design attributes under control of the investigator. As well, when considering the relative resource requirements of the designs, the costs listed in Online Table 11 must be considered, although formal cost comparison of designs is complex given that typically these costs will vary by site for multi-site trials, and over study time.

Our simulations made simplifying assumptions by necessity. For example, we assumed that the rate and timing of PrEP uptake is independent of baseline HIV risk and latent PrEP adherence groups. How a dependency would affect design performance is difficult to predict. For simplicity, we also assumed a time-constant vaccine efficacy, whereas in reality vaccine trials commonly anticipate and accommodate ramping efficacy (prior to full immunity) and waning efficacy given the limited durability of vaccine-induced immune responses. Since this ramping and waning is likely independent of PrEP use, we expect that the simplification does not influence the relative performance of the designs.

There are variations on the Run-In Designs that merit further investigation. As mentioned above, Run-In Designs that proceed to follow participants who continue on PrEP post-run-in for incident HIV infection have the merit that additional secondary objectives can be assessed around PrEP effectiveness and Vaccine vs. PrEP effectiveness. Alternatively, Run-In Designs may provide the vaccine to all or a random subset of participants who continue on PrEP post-run-in, in order to collect safety and immunogenicity outcomes among PrEP users. This may aid in licensure decisions and facilitate rollout of vaccine programs in populations using PrEP. As well, Run-In Designs may employ PrEP adherence monitoring throughout the duration of the run-in period(s), not just at the end of each period. While additional resources would be required for this more frequent adherence monitoring, there may be an advantage in terms of increased power and shorter average follow-up times as participants are randomized earlier in time. More generally, the length and number of the run-in periods require optimization from both a statistical and operational vantage-point, and need to be informed by anticipated PrEP uptake and adherence patterns.

The Run-In Designs we explored are similar to Sequential Multiple Assignment Randomized Trial (SMART) Designs which have been used to evaluate behavioral interventions that require modification under lack of adherence or response to intervention [1, 10, 26, 29–31]. SMART designs are appealing in that they reflect the need for certain interventions to be modified based on response, use, or tolerability. As well, they can provide data to discover optimal individual treatment policies. If in the future there are multiple PrEP variations for individuals to consider, a SMART design could be employed to compare the effectiveness of PrEP-A vs. PrEP-B, to evaluate vaccine efficacy, and also to discover an optimal treatment policy that combines vaccine and PrEP and takes into account subject preferences and adherence to PrEP.

The HIV prevention package continues to expand. Recent results from a phase 3 efficacy trial of injectable PrEP (Cabotegravir) suggest high efficacy relative to oral PrEP (tenofovir disoproxil fumarate/emtricitabine) in MSM and transgender women in North and South America, Asia, and southern Africa [32]. Another trial of the same intervention in women is ongoing. While there remain uncertainties about the long-term safety profile and acceptability of injectable PrEP, a likely scenario is that this intervention will be added to the HIV prevention package at some point in the near future. The designs discussed herein could have application to this context, as long as there remains a subpopulation of individuals who decline or are not able to adhere to either oral or injectable PrEP. Importantly, however, for the designs to be feasible, these individuals would need to be willing to be randomized to receive a vaccine. This may be unlikely; whereas oral PrEP and vaccines are different experiences for the participant (dosing, mode of delivery, side effects), injectable PrEP and vaccines provide more similar participant experiences, e.g., the HPTN 083 regimen entails an injection every 8 weeks and current HIV vaccine regimens involve 3–6-monthly injections. On the other hand, the current injectable PrEP regimen entails combining injections with oral PrEP; when an individual is taken off the injections, oral PrEP is given to “cover the tail”, i.e., to prevent HIV infection during a period when drug resistance could occur. Therefore, individuals who refuse oral PrEP currently are not eligible for injectable PrEP. Population acceptability of injectables, and future research on covering the tail of injectables, may modify these considerations.

While explored for the vaccine context, the designs we discussed may have application to other HIV prevention interventions that are viewed as alternatives to oral PrEP. It is recognized that many factors influence individual decision-making around products and practices to protect against HIV, and much like the field of contraception, multiple products will ultimately be needed to provide all at-risk populations with strategies that are effective, acceptable, and available [14]. Products such as vaginal rings, rectal microbicides, and other on-demand products will face similar challenges for efficacy trial design as for vaccines, and the designs we describe have direct application.

## Supplementary Material

Suppl Materials

## Figures and Tables

**Table 1:** Primary vaccine efficacy (VE) objectives and key secondary objectives addressed by each of the proposed designs

	Primary objective	Key secondary objectives

All-comers design	Evaluate VE in population not on stable PrEP	1. Evaluate VE among those not on PrEP at acquisition^a^
1-Stage Run-In Design	Evaluate VE in population declining or not adherent to PrEP at screening or after 1 stage of PrEP	1. Evaluate VE among those not on PrEP at acquisition^a^ 2. Evaluate PrEP effectiveness (PrEP vs. Placebo)^b^ 3. Compare Vaccine vs. PrEP effectiveness^b^
3-Stage Run-In Design	Evaluate VE in population declining or not adherent to PrEP at screening or after 1, 2, or 3 stages of PrEP	1. Evaluate VE among those not on PrEP at acquisition* 2. Evaluate PrEP effectiveness (PrEP vs. Placebo)^b^ 3. Compare Vaccine vs. PrEP effectiveness^b^
Decliners Design	Evaluate VE in population declining PrEP at screening	1. Evaluate VE among those not on PrEP at acquisition^a^

a Addressed using retrospective assaying of stored blood to test for ARV drug levels

b Evaluable if designs continue to follow and collect HIV endpoint data on individuals who remain interested and adherent to PrEP after each run-in period

**Table 2:** Simulation model parameters and assumed values for primary analyses

Parameter	Value	Description

*λ*_0_	3% per year, i.e., $1-e^{-\lambda_{0}*12\;\text{month}\;}=3\%$	Average HIV infection hazard, constant in time
*W* ∈ (1, 2, 3)	*W* ∼ *multinomial*(1/3, 1/3, 1/3)	Latent HIV risk group
*HR_W_*	(0.5, 1.0, 2.0)	Hazard ratio for each risk group, relative to average
*A* ∈ (1, 2, 3)	*A* ∼ *multinomial*(0.4, 0.3, 0.3)	Latent oral PrEP adherence group based on HPTN 069
*ρ_AW_*	*ρ_AW_* ∈ {−0.5, 0, 0.5}	Correlation coefficient between latent *A*, *W*
*t* ^uptake^	(0, 3, 6, ..., *T*) months post-enrollment or > maximum follow-up time *T*	PrEP uptake time, at screening (*t*^uptake^ = 0), during follow-up, or no uptake during follow-up (*t*^uptake^ > *T*)
*P*(*t*^uptake^ = *t*)	*P*(0) = 0.5, *P*(*t*^uptake^ > *T*) = 0.2, *P*(*k* * (3*m*)) = 0.3/14	50% uptake at screening, 20% no observed uptake, 30% uniform uptake across follow-up visits
*μ*(*t*)|*A* = *a*, *t*^uptake^	$I_{t>t^{\text{uptake}}\;}\left[\mu_{0a}+\beta_{1a}\left(\left(t-t^{\text{uptake}\;}\right)\right]\wedge t_{0a}\right)$ $+I_{t>t^{\text{uptake}}\;}[\beta_{2a}\left(t-t^{\text{uptake}\;}\right)I_{(t-t^{\text{uptake}}\;)>t_{0a}}]$ ^a^	Mean latent PrEP adherence for day *t* for those who take up PrEP, i.e., fraction of pills taken, based on HPTN 069
*σ*(*t*)|*A* = *a*	$\sigma (t)\sim N\left(0,\sigma_{a}^{2}\right),E\left(\sigma_{a}\right)=0.15$	Noise process of latent PrEP adherence
σ*(*t*)|*A* = *a*	$\sigma^{*}(t)\sim N\left(0,\sigma_{a}^{*2}\right),\sigma_{a}^{*}=2\sigma_{a}$	Noise process of measured PrEP adherence
*A*_0_	0.15 (corresponds to estimated PrEP hazard ratio of 0.46 based on Fig. 3, approximately 1 pill/week)	Threshold on fraction of pills taken per week Defining “adequate” oral PrEP adherence
*HR_PrEP_*(*A*(*t*))	$HR_{PrEP}(A(t))=e^{-7.21A{\left(t\right)}^{1.18}}$	PrEP hazard ratio given latent adherence *A*(*t*)
*Z*	*Z* ∼ *binomial*(0.5)	Randomly assigned treatment group
*VE*	*H*_0_ : *VE* = 0.25 and *H_a_* : *VE* ≥ 0.5	Vaccine efficacy, one minus hazard ratio
*λ_c_*	10% per year, i.e., $1-e^{-\lambda_{c}*12\;\text{month}\;}=10\%$	Non-informative censoring rate

a Parameter values for the linear change-point model can be found in Online Resources. Online Resources show results for additional parameter values

**Table 3:** Expected proportions of participants randomized at enrollment (*t* = 0) and after up to three 6-month Run-In periods, as a function of PrEP uptake and adherence

		*t* = 0	*t* = 6*m*^†^	*t* = 12*m*^‡^	*t* = 18*m*^‡^

All-Comers Design					
Decline PrEP at screening (*t*^uptake^ > 0)		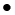	–	–	–
Uptake PrEP at screening (*t*^uptake^ = 0)		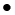	–	–	–
Run-In Designs					
Decline PrEP at screening (*t*^uptake^ > 0)		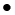	–	–	–
Uptake PrEP at screening (*t*^uptake^ = 0)					
	Consistently high adherence (*A* = 1)	–	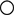	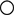	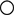
	Slowly declining adherence (*A* = 2)	–	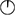	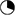	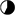
	Rapidly declining adherence (*A* = 3)	–	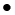	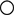	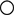
Decliners design					
Decline PrEP at screening (*t*^uptake^ > 0)		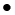	–	–	–
Uptake PrEP at screening (*t*^uptake^ = 0)		–	–	–	–

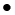
 > 99% randomized

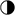

some portion randomized◇

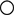
 randomized with probability near zero

– not eligible for randomization

Fully filled black circles indicate that nearly all participants are randomized that time. Partially filled black circles show the expected proportions of participants randomized. Dash lines mean in that design no randomization is done at the time

† Randomized after first 6-month run-in period for 1-Stage and 3-Stage Run-In Designs

‡ Randomized after second or third run-in periods under the 3-Stage Run-In Design

◇ Black fill amount corresponds to the expected proportion randomized

**Table 4:** Required number to randomize to Vaccine vs. Placebo (*N_R_*), and number enrolled (*N_E_*) and offered PrEP at screening (*N_PS_*), to achieve 90% empirical power to detect *H_a_* : *VE* = 50% vs. *H*_0_ : VE = 25%, controlling 2-sided *α* = 0.05, for the four proposed designs

Design	*ρ_AW_*	*N_PS_*	*N_E_*	$N_{R}(N_{R_{k}})$	$N_{R}/N_{R}^{\text{All-Comers}}(\%)$

All-Comers	−0.5	10,500	10,500	10,500	100
	0	9000	9000	9000	100
	0.5	8500	8500	8500	100
1-Stage Run-In	−0.5	11,800	11,800	7124 (5900, 1224)	67.8
	0	11,200	11,200	6762 (5600, 1162)	75.1
	0.5	10,500	10,500	6338 (5250, 1088)	74.6
3-Stage Run-In	−0.5	10,000	10,000	7647 (5000, 1039, 747, 861)	72.8
	0	9500	9500	7265 (4750, 987, 710, 818)	80.7
	0.5	8500	8500	6491 (4250, 880, 631, 730)	76.4
Decliners	−0.5	15,000	7500	7500	71.4
	0	14,000	7000	7000	77.8
	0.5	13,600	6800	6800	80.0

Numbers are shown for scenarios with correlation between latent adherence and HIV risk categories of *ρ_AW_* = −0.5, 0 and 0.5. For the Run-In Designs, the numbers randomized at enrollment and after each of the run-in periods are shown
