# Supplementary material for: Designing HIV Vaccine Efficacy Trials in the Context of Highly Effective Non-vaccine Prevention Modalities
Source: Stat Biosci. Author manuscript; Available in PMC 2023 Mar 17. (PMC10022814; doi:10.1007/s12561-020-09292-1)
Supplement: Suppl Materials [file NIHMS1834234-supplement-Suppl_Materials.pdf]

Online Resources

Parameter values used to simulate PrEP adherence trajectories

Using data from HPTN 069, oral PrEP pill taking patterns based on Wisepill monitoring were modeled with a linear change-point model with mean process $\mu_a(t) = \mu_{0a} + \beta_{1a}(t \wedge t_{0a}) + \beta_{2a}tI(t > t_{0a})$ and additive random noise process $\sigma(t) \sim N(0, \sigma_a^2)$, for adherence category $a = 1,2,3$ [42]. Here, t indexes day and t_{0a} is the adherence-category-specific change-point. The maximum likelihood estimates of model parameters $(\hat{\mu}, \hat{t}_0, \hat{\beta}_1, \hat{\beta}_2)$ based on HPTN 069 are: $(0.90, 200.0, 0.00, 0.00)$ for the "high" $(a = 1)$ adherence category, $(0.85, 150.0, -0.0014, -0.0019)$ for the "intermediate" $(a = 2)$ adherence category, and $(0.57, 82.1, -0.0038, -0.0020)$ for the "poor" $(a = 3)$ adherence category. Individual-level latent daily adherence trajectories $A_i(t)$ are simulated from this fitted linear change-point model, i.e. for $A_i = a$ the trajectory is drawn from the model with parameters $(\hat{\mu}_a, \hat{t}_{0a}, \hat{\beta}_{1a}, \hat{\beta}_{2a})$. Note that while HPTN 069 only followed participants for 12 months, the fitted model is used to extrapolate adherence trajectories through 24 months. Since the average standard deviation of the random noise σ_a's in HPTN 069 ranges from 0.1 to 0.2 depending on the smoothing pre-processing procedure, we assume $\sigma_a \sim N(0.15, 0.1)$ and the noise process for the individual-level latent adherence trajectory for adherence category $A = a$ is $\sigma(t) \sim N(0, \sigma_a^2)$. Furthermore, to account for additional uncertainty in measured adherence, we assume $A_i^{obs}(t)$ has noise process $\sigma^*(t) \sim N(0, 4 \cdot \sigma_a^2)$.

Results for different simulation model parameters

Table 5: Required number to randomize to Vaccine vs. Placebo (N_R), and number enrolled (N_E) and offered PrEP at screening (N_{PS}), to achieve 90% empirical power to detect $H_a : VE = 50\%$ vs. $H_0 : VE = 25\%$, controlling 2-sided $\alpha = 0.05$, for the four proposed designs. **Simulation model parameters are as in Table 2 except that the marginal HIV incidence is higher, at 4 per 100 person-years.** Numbers are shown for scenarios with correlation between latent adherence and HIV risk categories of $\rho_{AW} = -0.5, 0$ and 0.5. For the Run-In Designs, the numbers randomized at enrollment and after each of the run-in periods are shown.

Design	ρ_{AW}	N_{PS}	N_E	$N_R(N_{R_k})$	$N_R/N_R^{\text{All-Comers}}$
All-Comers	-0.5	8000	8000	8000	100
	0	7500	7500	7500	100
	0.5	7000	7000	7000	100
1-Stage Run-In	-0.5	9000	9000	5434 (4500, 934)	67.9
	0	8200	8200	4950 (4100, 850)	66.0
	0.5	7800	7800	4705 (3900, 805)	67.2
3-Stage Run-In	-0.5	7200	7200	5498 (3600, 745, 536, 617)	68.7
	0	7000	7000	5343 (3500, 725, 519, 599)	71.2
	0.5	6700	6700	5110 (3350, 691, 495, 574)	73.0
Decliners	-0.5	11000	5500	5500	68.7
	0	10800	5400	5400	72.0
	0.5	10400	5200	5200	74.3

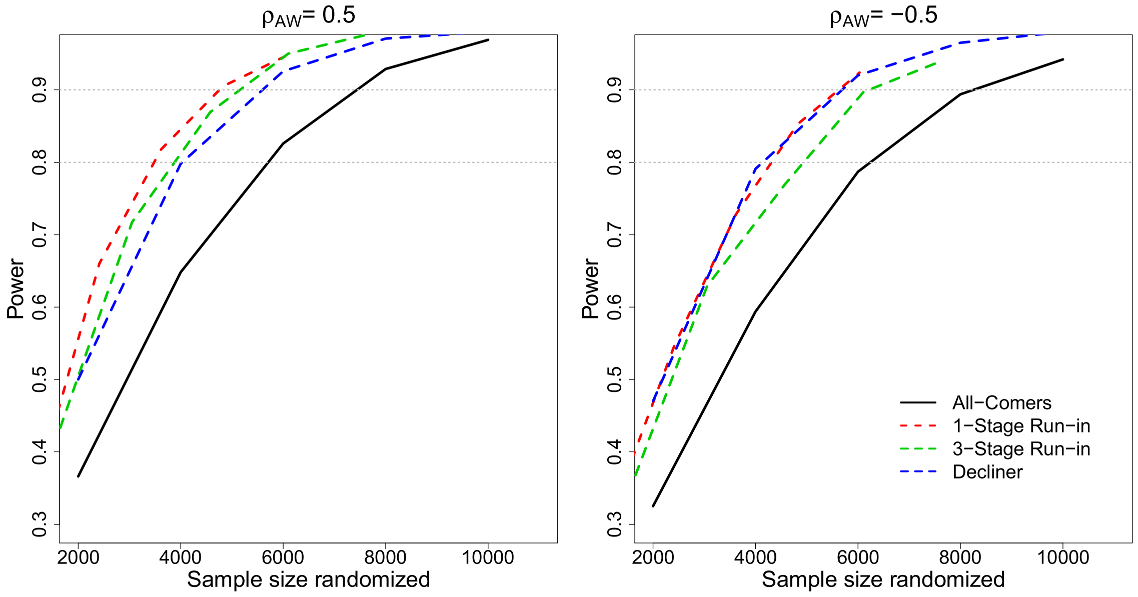

Fig. 7: Empirical power to detect $H_a : VE = 50\%$ versus $H_0 : VE = 25\%$ as a function of the total number of randomized participants (N_R) for each proposed study design. **Simulation model parameters are as in Table 2 except that the marginal HIV incidence is higher, at 4 per 100 person-years.** Power is based on 1000 simulations and a 2-sided 0.05-level log-rank test (stage-stratified for Run-In Designs), and is shown for a scenario in which low risk individuals tend to be more adherent to PrEP ($\rho_{AW} = 0.5$, left) and a scenario in which high risk individuals tend to be more adherent ($\rho_{AW} = -0.5$, right).

Table 6: Required number to randomize to Vaccine vs. Placebo (N_R), and number enrolled (N_E) and offered PrEP at screening (N_{PS}), to achieve 90% empirical power to detect $H_a : VE = 50\%$ vs. $H_0 : VE = 25\%$, controlling 2-sided $\alpha = 0.05$, for the four proposed designs. **Simulation model parameters are as in Table 2 except that the initial PrEP uptake rate at screening is lower,** $P(t^{uptake} = 0) = 0.25$**, and the fraction who never take up PrEP is higher,** $P(t^{uptake} > T) = 0.3$**.** Numbers are shown for scenarios with correlation between latent adherence and HIV risk categories of $\rho_{AW} = -0.5, 0$ and 0.5. For the Run-In Designs, the numbers randomized at enrollment and after each of the run-in periods are shown.

Design	ρ_{AW}	N_{PS}	N_E	$N_R(N_{R_k})$	$N_R/N_R^{\text{All-Comers}}$
All-Comers	-0.5	9000	9000	9000	100
	0	8300	8300	8300	100
	0.5	8000	8000	8000	100
1-Stage Run-In	-0.5	9500	9500	7618 (7125, 493)	84.6
	0	8500	8500	6816 (6375, 441)	82.1
	0.5	8000	8000	6415 (6000, 415)	80.2
3-Stage Run-In	-0.5	8000	8000	7056 (6000, 415, 298, 343)	78.4
	0	7800	7800	6881 (5850, 405, 291, 335)	82.9
	0.5	7500	7500	6616 (5625, 389, 280, 322)	82.7
Decliners	-0.5	10000	7500	7500	83.3
	0	9333	7000	7000	84.3
	0.5	9067	6800	6800	85.0

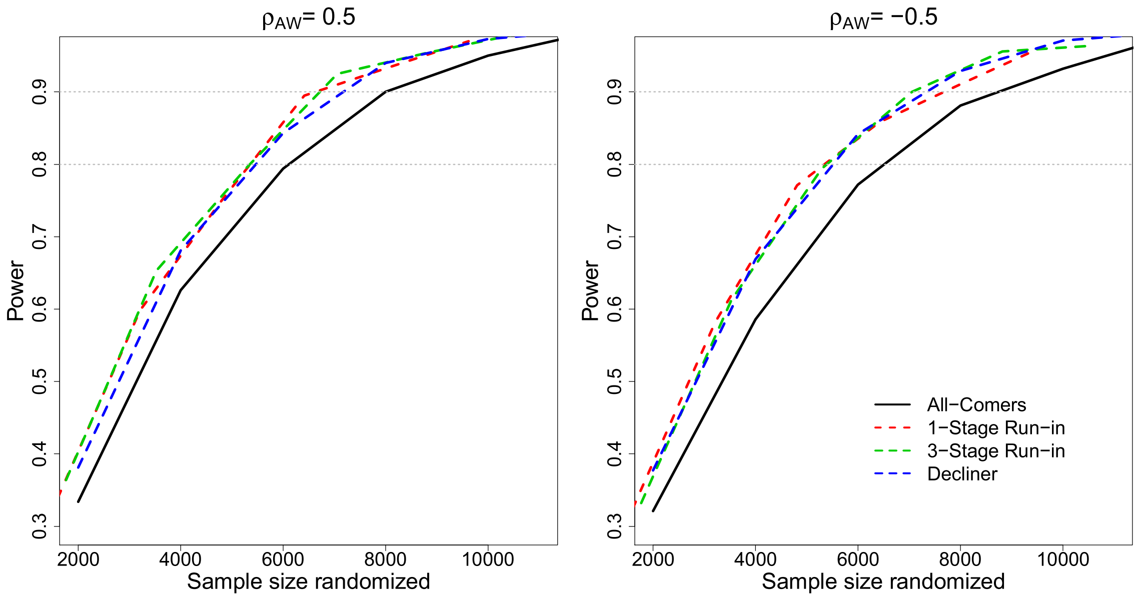

Fig. 8: Empirical power to detect $H_a : VE = 50\%$ versus $H_0 : VE = 25\%$ as a function of the total number of randomized participants (N_R) for each proposed study design. **Simulation model parameters are as in Table 2 except that the initial PrEP uptake rate at screening is lower,** $P(t^{uptake} = 0) = 0.25$**, and the fraction who never take up PrEP is higher,** $P(t^{uptake} > T) = 0.3$**.** Power is based on 1000 simulations and a 2-sided 0.05-level log-rank test (stage-stratified for Run-In Designs), and is shown for a scenario in which low risk individuals tend to be more adherent to PrEP ($\rho_{AW} = 0.5$, left) and a scenario in which high risk individuals tend to be more adherent ($\rho_{AW} = -0.5$, right).

Table 7: Required number to randomize to Vaccine vs. Placebo (N_R), and number enrolled (N_E) and offered PrEP at screening (N_{PS}), to achieve 90% empirical power to detect $H_a : VE = 50\%$ vs. $H_0 : VE = 25\%$, controlling 2-sided $\alpha = 0.05$, for the four proposed designs. **Simulation model parameters are as in Table 2 except that the adherence threshold that defines adequate PrEP adherence and determines randomization eligibility is higher,** $A_0 = 0.3$**.** Numbers are shown for scenarios with correlation between latent adherence and HIV risk categories of $\rho_{AW} = -0.5, 0$ and 0.5. For the Run-In Designs, the numbers randomized at enrollment and after each of the run-in periods are shown.

Design	ρ_{AW}	N_{PS}	N_E	$N_R(N_{R_k})$	$N_R/N_R^{\text{All-Comers}}$
All-Comers	-0.5	10500	10500	10500	100
	0	9000	9000	9000	100
	0.5	8500	8500	8500	100
1-Stage Run-In	-0.5	12000	12000	7200 (6000, 1200)	68.6
	0	11500	11500	6900 (5750, 1150)	76.7
	0.5	11000	11000	6600 (5500, 1100)	77.6
3-Stage Run-In	-0.5	10500	10500	7963 (5250, 1050, 1016, 647)	75.8
	0	9200	9200	6978 (4600, 920, 891, 567)	77.5
	0.5	8800	8800	6674 (4400, 880, 852, 542)	78.5
Decliners	-0.5	15000	7500	7500	71.4
	0	14000	7000	7000	77.8
	0.5	13600	6800	6800	80.0

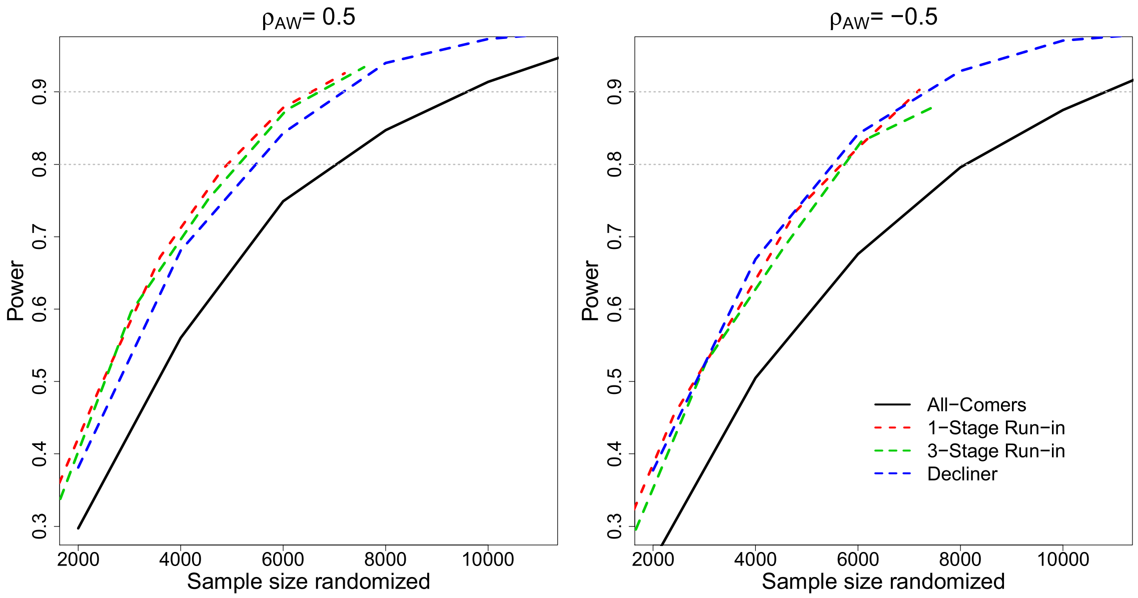

Fig. 9: Empirical power to detect $H_a : VE = 50\%$ versus $H_0 : VE = 25\%$ as a function of the total number of randomized participants (N_R) for each proposed study design. **Simulation model parameters are as in Table 2 except that the adherence threshold that defines adequate PrEP adherence and determines randomization eligibility is higher,** $A_0 = 0.3$**.** Power is based on 1000 simulations and a 2-sided 0.05-level log-rank test (stage-stratified for Run-In Designs), and is shown for a scenario in which low risk individuals tend to be more adherent to PrEP ($\rho_{AW} = 0.5$, left) and a scenario in which high risk individuals tend to be more adherent ($\rho_{AW} = -0.5$, right).

Table 8: Expected proportions of participants randomized at enrollment ($t = 0$) and after up to three 3-month Run-In periods, as a function of PrEP uptake and adherence **(in contrast to three 6-month run-in periods as in Table 3).** Fully filled black circles indicate that nearly all participants are randomized that time. Partially filled black circles show the expected proportions of participants randomized. Dash lines mean in that design no randomization is done at the time.

	$t = 0$	$t = 3m^{\dagger}$	$t = 6m^{\ddagger}$	$t = 9m^{\ddagger}$
**All-Comers Design**				
Decline PrEP at screening ($t^{uptake} > 0$)	●	—	—	—
Uptake PrEP at screening ($t^{uptake} = 0$)	●	—	—	—
**Run-In Designs**				
Decline PrEP at screening ($t^{uptake} > 0$)	●	—	—	—
Uptake PrEP at screening ($t^{uptake} = 0$)				
Consistently high adherence ($A = 1$)	—	○	○	○
Slowly declining adherence ($A = 2$)	—	◔	◔	◔
Rapidly declining adherence ($A = 3$)	—	◔	◕	○
**Decliners Design**				
Decline PrEP at screening ($t^{uptake} > 0$)	●	—	—	—
Uptake PrEP at screening ($t^{uptake} = 0$)	—	—	—	—

● : > 99% randomized ◑ : some portion randomized$^{\diamond}$

○ : randomized with probability near zero — : not eligible for randomization

†randomized after first 3 month run-in period for 1-Stage and 3-Stage Run-In Designs

‡randomized after second or third run-in periods under the 3-Stage Run-In Design

$^{\diamond}$black fill amount corresponds to the expected proportion randomized

Table 9: Required number to randomize to Vaccine vs. Placebo (N_R), and number enrolled (N_E) and offered PrEP at screening (N_{PS}), to achieve 90% empirical power to detect $H_a : VE = 50\%$ vs. $H_0 : VE = 25\%$, controlling 2-sided $\alpha = 0.05$, for the four proposed designs. **Simulation model parameters are as in Table 2 except that the run-in period duration is 3 months instead of 6 months.** Numbers are shown for scenarios with correlation between latent adherence and HIV risk categories of $\rho_{AW} = -0.5, 0$ and 0.5. For the Run-In Designs, the numbers randomized at enrollment and after each of the run-in periods are shown.

Design	ρ_{AW}	N_{PS}	N_E	$N_R(N_{R_k})$	$N_R/N_R^{\text{All-Comers}}$
All-Comers	-0.5	10500	10500	10500	100
	0	9000	9000	9000	100
	0.5	8500	8500	8500	100
1-Stage Run-In	-0.5	13000	13000	7020 (6500, 520)	66.9
	0	14500	14500	7830 (7250, 580)	87.0
	0.5	14000	14000	7560 (7000, 560)	88.9
3-Stage Run-In	-0.5	12500	12500	8088 (6250, 500, 938, 400)	77.1
	0	10000	10000	6470 (500, 400, 750, 320)	71.9
	0.5	9500	9500	6147 (4750, 380, 713, 304)	72.3
Decliners	-0.5	15000	7500	7500	71.4
	0	14000	7000	7000	77.8
	0.5	13600	6800	6800	80.0

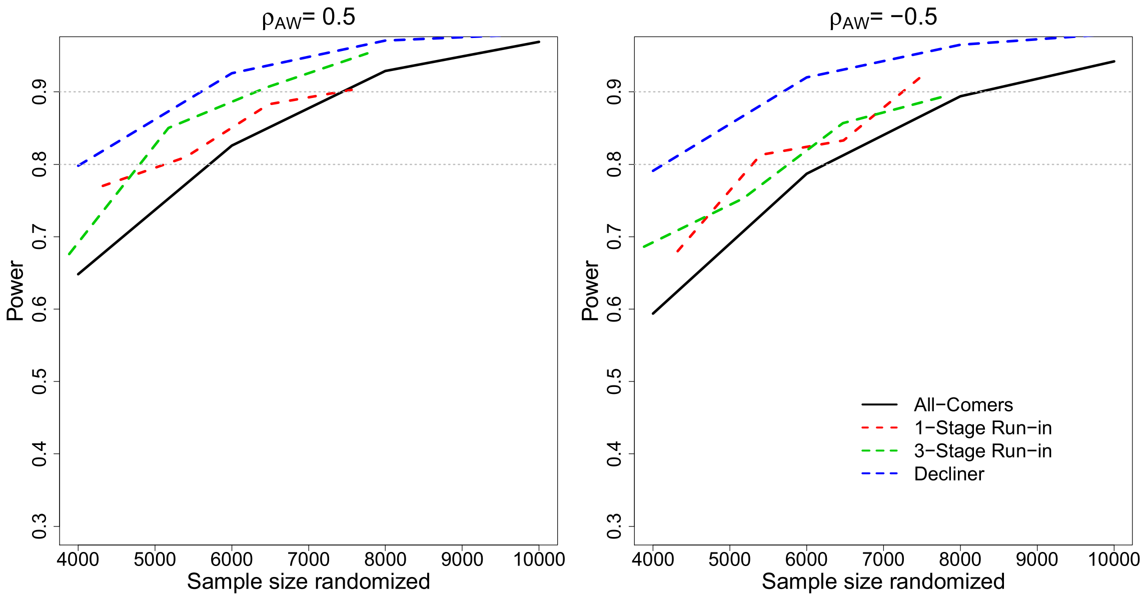

Fig. 10: Empirical power to detect $H_a : VE = 50\%$ versus $H_0 : VE = 25\%$ as a function of the total number of randomized participants (N_R) for each proposed study design. **Simulation model parameters are as in Table 2 except that the run-in period duration is 3 months instead of 6 months.** Power is based on 1000 simulations and a 2-sided 0.05-level log-rank test (stage-stratified for Run-In Designs), and is shown for a scenario in which low risk individuals tend to be more adherent to PrEP ($\rho_{AW} = 0.5$, left) and a scenario in which high risk individuals tend to be more adherent ($\rho_{AW} = -0.5$, right).

Table 10: Attributes of target population, clinical context, and trial design, and relevance for gauging relative power of designs. While design attributes are modifiable, i.e. under the control of the investigator, attributes of the target population and the clinical context are not.

	Attribute of Population, Context, or Design?	Major impact on relative power?	Varied in simulations?
Avg. placebo HIV incidence absent PrEP, assumed constant	Population	No	Yes
Heterogeneity in placebo HIV incidence absent PrEP	Population	Unlikely	No
Latent PrEP adherence groups, profiles of, variability within, and correlation with HIV risk category	Population	Yes	Yes*
PrEP uptake rate, assumed indep. of HIV risk category and PrEP adherence	Population	Yes	Yes+
Length of run-in period	Design	Yes	Yes
PrEP adherence randomization threshold	Design	Yes	Yes
PrEP efficacy as a function of adherence	Context	Yes	No
Vaccine efficacy, assumed constant	Context	No	No
Randomization fraction	Design	Unlikely	No
Independent censoring rate	Context	Unlikely	No

* Correlation between latent PrEP adherence group and HIV risk group was varied, but frequencies and profiles of adherence groups were held fixed.

+ PrEP uptake rate was varied, but throughout uptake was assumed to be independent of adherence and HIV risk.

Table 11: Costs of study procedures that will influence the relative resource requirements of the designs. Costs are likely to vary among sites, for multi-site trials, and may vary over the course of the study.

Cost of screening an individual for PrEP interest
Cost of enrolling an individual
Cost of providing PrEP to an individual (product and associated clinical management), per unit time
Cost of PrEP adherence evaluation for an individual (specimen collection, processing, storage, assay), per evaluation
Cost of randomizing and following an individual participant (Vaccine/Placebo administration; study visits; specimen collection, processing, storage, assays; endpoint determination)
